# Not all sitting is equal: Novel neuroimaging insights into mentally passive versus active sedentary behaviors

**DOI:** 10.1002/alz.71905

**Published:** 2026-10-13

**Authors:** Mats Hallgren, André Werneck, Liye Zou, David W Dunstan, Neville Owen

**Affiliations:** ^1^ Deakin Institute for Physical Activity and Nutrition Deakin University Melbourne Australia; ^2^ Department of Global Public Health Science Karolinska Institute Stockholm Sweden; ^3^ Center for Epidemiological Research in Nutrition and Health University of São Paulo São Paulo Brazil; ^4^ Department of Kinesiology's Metabolism Nutrition and Exercise Laboratory University of Wisconsin–Madison Madison Wisconsin USA; ^5^ Body‐Brain‐Mind Laboratory School of Psychology Shenzhen University Shenzhen China; ^6^ Wuhan Sports University Wuhan China; ^7^ Baker Heart and Diabetes Institute Melbourne Australia; ^8^ School of Health Sciences Swinburne University of Technology Melbourne Australia

1

The recent study by Feter and colleagues^1^ provides a vital contribution to the understanding of lifestyle factors and structural brain aging. Clinical and public health guidelines have largely focused on physical activity and overall sedentary time, with limited consideration of the cognitive and social context in which sitting occurs.^2^ Importantly, using structural magnetic resonance imaging (MRI) data from the Atherosclerosis Risk in Communities (ARIC) cohort, Feter and colleagues demonstrate that the neurological correlates of sitting can be context‐dependent and sex‐specific.^1^ Frequent television viewing was associated with increased white matter hyperintensity (WMH) volume and reduced cortical volumes, while more occupational sitting was associated with larger frontal volumes.^1^ These new findings expand the broadening base of evidence that sedentary‐behavior type and context – not only total sitting duration—may moderate associations with cognitive, structural, and functional brain outcomes.^3^

Use of the well‐characterized ARIC cohort,^3^ evaluating midlife sedentary habits and mapping them to 3T structural MRI metrics over a long follow‐up period, provides a prospective link between midlife sedentary habits and later‐life structural brain measures. The focus on Alzheimer's disease related regions and WMHs provided a granular assessment of neurodegenerative and cerebrovascular pathologies.^1^

Crucially, relevant covariates were controlled for, including education, physical activity, diabetes status, body mass index, smoking, and alcohol use.^1^ The persistence of these associations after adjustment suggests that the findings are unlikely to be attributable to confounding or lack of moderate‐to‐vigorous physical activity.^4^ Adverse structural changes and protective occupational correlates were observed in male participants.^1^ The authors note that variations might stem from different sitting habits or biological differences in brain aging. Because the evaluation of sedentary behavior relied on categorical self‐reported recall over the preceding 12 months,^1^ and without device‐based activity data, it is difficult to determine exact thresholds associated with the structural modifications. Moreover, without precise data on sitting patterns and the frequency of physically active interruptions, these hypotheses remain compelling but speculative. Although the long follow‐up minimizes reverse causality, subclinical neurodegenerative changes or executive dysfunction in midlife might influence preferences for passive leisure activities, rather than the reverse.

The findings of Feter and colleagues provide a welcome and powerful complement to recent epidemiological studies on depression and dementia.^4^, ^5^, ^6^, ^7^ For example, in a 19‐year follow‐up of over 20,000 individuals, we differentiated between mentally passive (e.g., TV‐viewing) and mentally active (e.g., reading) sedentary behaviors.^4^ Higher volumes of mentally passive sitting significantly increased risk of incident dementia and depression.^4^, ^7^ Conversely, mentally active sitting was associated with lower risk.^4^, ^7^ The new ARIC study findings provide convergent evidence on plausible structural correlates of the epidemiological observations.^1^ Smaller frontal lobe volumes and elevated white matter damage among heavy TV viewers may help to explain a higher clinical trajectory toward dementia. Furthermore, the finding that cognitively active occupational sitting was associated with frontal cortex volume and cerebrovascular health may explain why mentally active sitting can help to support cognitive reserve.^1^ This alignment across independent cohorts—epidemiological registries in Sweden^4^, ^7^ and neuroimaging cohorts in the United States^1^—strongly supports the hypothesis that lower levels of brain activity during sedentary behavior may contribute to long‐term adverse neurological impacts.

Studies pairing high‐resolution accelerometry with real‐time ecological momentary assessments could help to identify whether the protective effect of mentally active sitting is driven by cognitive stimulation, or if it is a byproduct of frequent postural transitions common in office environments. The dual‐axis taxonomy combining mental activation with content relevance^8^ provides a potential framework to advance such studies, which could integrate fluid and neuroimaging biomarkers, such as amyloid‐beta and markers of neuroinflammation, to investigate how mentally passive sitting can influence brain pathology.

In a contemporary context where screen‐based entertainment is ubiquitous and proliferating, determining whether prolonged uninterrupted mentally passive sitting accelerates vascular brain disease via WMHs could provide strong support for the targetting of clinical and preventive strategies.^9^ To gather further informative findings, portable functional near‐infrared spectroscopy (fNIRS) may complement structural MRI by capturing task and context‐sensitive cerebral hemodynamics during sedentary activities. Future studies will need sedentary‐behavior–specific standards for physiological‐confound monitoring, quality control, and multiple‐comparison correction.^10^ A plethora of exciting scientific opportunities exists in this space.

With replication and supported by intervention trial evidence, these welcome new findings from Feter and colleagues will contribute strongly to more context‐sensitive clinical and public‐health guidance for improving brain health and wellbeing.

## FUNDING INFORMATION

The authors have nothing to report.

## CONFLICT OF INTEREST STATEMENT

The authors declare no conflicts of interest. Author disclosures are available in the Supporting Information.

## Supporting information




Supporting Information
